# Leading Innovative Work-Behavior in Times of COVID-19: Relationship Between Leadership Style, Innovative Work-Behavior, Work-Related Flow, and IT-Enabled Presence Awareness During the First and Second Wave of the COVID-19 Pandemic

**DOI:** 10.3389/fpsyg.2021.717345

**Published:** 2021-09-28

**Authors:** Martine J. H. Coun, Robin Edelbroek, Pascale Peters, Robert J. Blomme

**Affiliations:** ^1^Faculty of Management, Open Universiteit, Heerlen, Netherlands; ^2^Strategy, Organization and Leadership Centre, Nyenrode Business Universiteit, Breukelen, Netherlands

**Keywords:** innovative work-behavior, empowering leadership, directive leadership, work-related flow, IT-enabled presence awareness, COVID-19 pandemic, telework, remote working

## Abstract

**Aim:** The present study contributes to the conversation on remote (home) working, leadership, and innovation in times of COVID-19 by examining the mediating role of work-related flow in the relationship between empowering and directive leadership, on the one hand, and innovative work-behavior, on the other, and the moderating role of IT-enabled presence awareness in two lockdown periods during the pandemic.

**Method:** We employed PLS-SEM analysis to analyze the perceptions, experiences, and behaviors of a group of employees (*N* = 257) regarding the study’s core variables during two phases of the COVID-19 pandemic (summer 2020 and autumn 2020).

**Results:** In line with expectations, in the earlier phase of the pandemic, empowering leadership had both a positive direct and indirect relationship with innovative work-behavior via work-related flow, whereas directive leadership only had a negative direct relationship with innovative work-behavior. In the second phase, however, empowering leadership only had a positive indirect relationship with innovative work-behavior, running via work-related flow. Moreover, directive leadership was both directly and indirectly negatively related to innovative work-behavior, via work-related flow. In contrast to our expectations, IT-enabled presence awareness did not play a moderating role in these relationships in any phase.

**Discussion:** Our findings underline the importance of empowerment in sustaining innovative work-behavior, particularly in intense and enduring remote work contexts, as this can amplify employees’ ability, motivation and opportunity to generate, share and implement novel ideas. In remote work contexts, empowering leadership can particularly foster innovation indirectly via work-related flow, which was also shown to be an increasingly important underlying mechanism across time periods. Directive leadership, in contrast, can reduce work-related flow and, therefore, hinder innovation. Our study did not find evidence for the moderating role of employees’ perceptions of IT-enabled presence awareness.

**Conclusion:** We conclude that regardless of the IT-quality, the leadership style chosen plays an important role in innovative work-behavior in remote work-contexts, particularly in view of the divergent effects of empowering and directive leadership on work-related flow in enduring and intense remote work contexts.

## Introduction

In compliance with the social-distancing regulations imposed by national governments to avoid the spread of the COVID-19-virus, many employees continued their regular work activities while working remotely using information and communication technologies (IT). The sudden shift toward homeworking forced many organizations to improvise and to develop new work routines to virtually serve customers and to collaborate with others inside and outside the organization. This shift also demanded employees to engage in *innovative work-behaviors* (Janssen, 2000) to make the best of the situation and to even flourish in the rapidly changing work environment. According to Janssen (2000), innovative work-behavior can be defined “as the intentional creation, introduction and application of new ideas within a work role, group, or organization, in order to benefit role performance, the group, or the organization” (p. 228). Innovative work-behavior, however, may not be that easy in remote work-contexts, as employees’ reliance on technology to facilitate their collaboration increases (Gibson and Gibbs, 2006).

Leadership is shown to be a crucial factor in innovative work-behavior as leaders shape the working environment, allocate resources, and influence employees’ innovative work-behaviors by controlling, motivating, and inspiring them (Lee A. et al., 2020). The importance of leadership may even be amplified by the current COVID-19 pandemic, as employees who are forced to work virtually might not know how to act and need guidance to adapt to the new work-situation (Carnevale and Hatak, 2020; Montani and Staglianò, 2021). The question, therefore, arises how leaders have responded to the new situation, as virtual working may have changed the relationships between leaders and employees, and among employees, since it is harder to control and motivate employees directly (Peters et al., 2016).

Not all leaders may have adapted similarly to the changing work conditions following the COVID-19 pandemic (Bajaba et al., 2021). Some leaders may have seized the opportunity to allow their employees to raise ideas to find solutions for occurring problems and may have shifted decision-making power, autonomy, and accountability to their employees by embracing *empowering leadership* (Ahearne et al., 2005). The increased job autonomy that employees may experience resulting from this change in leadership behavior may have helped them to explore novel and creative ideas and enhance innovative work-behaviors (Lee A. et al., 2020). Other leaders, however, may have shifted to micromanagement to compensate for the loss of direct control in virtual work-settings, as this resolves ambiguity and uncertainty among leaders and employees and provides clear guidelines (Stoker et al., 2019). In contrast to empowering leadership, *directive leadership* centralizes decision making, which implies that the formal leader issues instructions and commands to employees and assigns collective goals (Pearce and Sims, 2002).

Up until now, however, it is not clear which leadership response is best in crisis situations, such as the COVID-19-pandemic. It can be argued that directive leadership, in response to a crisis, might prove to be effective in the short run, but can also be detrimental to innovative work-behavior in the longer run (Somech, 2005; Stoker et al., 2019). Given that the COVID-19 pandemic presents an unprecedented challenge to managing today’s workforce, there is a knowledge gap in the extant literature on how and to which extent empowering and directive leadership influence employees’ perceptions of their innovative work-behavior. In a similar vein, there is a lack of insight into the extent to which these relationships change (Lee A. et al., 2020) after a prolonged time of working remotely. Related to that, it can be questioned what the underlying mechanism is that links different leadership approaches to innovative work-behavior, and to what extent the relationships with this mechanism changes in strength over time.

Regarding the underlying mechanism, it can be pointed out that innovative work-behavior is highest when employees enjoy their work, are intrinsically motivated, and are fully absorbed in it (Maqbool et al., 2018). The state of consciousness that fits these three conditions can be referred to as *flow* (Csikszentmihalyi, 1975). Applied to work situations, this is referred to as *work-related flow* (Bakker, 2008). It is not clear, however, whether and to what extent both directive leadership and empowering leadership can foster innovative work-behavior via enhanced work-related flow and how this potential mediating role of work-related flow differs across different periods of time in remote work situations during the COVID-19-pandemic.

Moreover, the mediating role of work-related flow in the relationship between leadership and innovative work-behavior may be contingent on the quality of communication between employees with the leader and with peers. The lack of physical co-presence in the case of homeworking may indirectly have consequences for employees’ innovative work-behavior (Gibson and Gibbs, 2006). More specifically, it is known that IT-mediated communication during homeworking tends to hinder the communication richness in comparison with face-to-face communication (Martins et al., 2004). With modern technologies, however, the quality of digital communication may come close to that of face-to-face communication. This implies that employees’ perceptions of so-called *IT-enabled presence awareness* (Malhotra and Majchrzak’s, 2012, 2014; Lim, 2018), that is, the degree to which the quality of virtual communication is perceived by employees to equal that of face-to-face communication, can be an important boundary condition affecting the relationship between leadership and work-related flow. Moreover, it is not clear whether the moderating role of perceived IT-enabled presence awareness in the relationship between leadership and work-related flow is affected by the duration of the COVID-19-regulations that demand employees to work from home.

In view of the gaps presented above, the present study aims to contribute to the conversation on leadership and innovation by examining the mediating role of work-related flow between empowering and directive leadership and innovative work-behavior in times of COVID-19, and the moderating role of IT-enabled presence awareness on the relationship between leadership and work-related flow. Furthermore, we examine possible differences herein between two time periods during the COVID-pandemic that vary regarding the duration and intensity of homeworking. The contributions of analyzing our moderated mediation model to the extant literature are multiple: First, we contribute to the conversation on the relationship between leadership and innovative work-behavior by investigating two leadership styles: empowering and directive leadership (Lorinkova et al., 2013). Second, we examine the change in these relationships across time, which is especially interesting in times of the COVID-19 pandemic in which homeworking policies are constantly prolonged (Bajaba et al., 2021). In view of this, we investigate the influence of empowering and directive leadership on individual employees’ innovative work-behavior at two moments in time (Lee A. et al., 2020). Third, we add to the literature on work-related flow (Bakker, 2008) by investigating the potential mediating role of work-related flow in the relationship between empowering and directive leadership, and innovative work-behavior. Fourth, by comparing two episodes in the COVID-19 pandemic, we examine the influence of the mediating role of work-related flow in these relationships in the context of working from home. Fifth, we contribute to literature on home-based working (Martins et al., 2004; Peters et al., 2016) and leadership (Cortellazzo et al., 2019) by investigating the potential moderating role of IT-enabled presence awareness (Lim, 2018) on the relationships between empowering and directive leadership, on the one hand, and work-related flow on the other. Sixth, by comparing two episodes in the COVID-19 pandemic, we examine whether the influence of IT-enabled presence awareness is contingent on the duration and intensity of the homeworking practice in which routines, cognitions, and behaviors may have changed.

This study is structured as follows. Based on the literatures on innovative work-behavior, leadership, work-related flow, and IT-enabled presence awareness, a set of hypotheses is developed. Subsequently, the study’s data and methodology are outlined. Then, the results of the study are presented and, subsequently, discussed in the light of our theoretical framework and methodology. In conclusion, the study’s limitations and implications for scholarly research and management practice are presented.

## Theory and Hypotheses

### Innovative Work-Behavior During the COVID-19 Pandemic

The COVID-19 pandemic has completely shifted the circumstances in which organizations operate. An organization’s capability to innovate is, therefore, particularly important during this time, not only to ensure its short-term survival, but also its long-term positioning (Montani and Staglianò, 2021). Innovation on an organizational level is largely driven by the innovative work-behavior of its own employees (Liu et al., 2017) and their perceptions thereof (Janssen, 2000). Innovative work-behavior consists of three behavioral tasks: idea generation, idea promotion, and idea realization (Janssen, 2000). However, the restrictions and stringencies that have been imposed by national governments to avoid the risk of infection with the coronavirus may have affected employees’ innovative work-behaviors (Kapoor et al., 2021). Aside from the communication challenges that arise resulting from remote working (Martins et al., 2004), employees may feel uncertain due to the changes caused by the pandemic which may have hampered their innovative work-behavior (Montani and Staglianò, 2021).

### Empowering Leadership and Innovative Work-Behavior

In the current COVID-19 pandemic, many leaders are challenged to engage with their employees remotely and create a supportive working environment that allows them to thrive (Contreras et al., 2020). Empowering leaders seek to achieve this through enhancing employees’ levels of job autonomy and responsibility by sharing information about the organizational direction and the meaningfulness of the employee’s work therein, while involving them in decision making (Lorinkova et al., 2013; Martin et al., 2013). According to Sardeshmukh et al. (2012), one of the primary benefits of remote working is increased job autonomy, which enhances employees’ work engagement. Once employees experience more meaningfulness, autonomy and decision latitude, this may also benefit their innovative work-behavior (Scott and Bruce, 1994; Amabile and Pratt, 2016). In a similar vein, Cheong et al. (2019) notes that empowering leadership aims to enhance employees’ development of higher competencies and confidence in their own abilities. Due to this, employees may feel motivated to freely explore new ideas and increased engagement in creative processes (Zhang and Bartol, 2010). Rao Jada et al. (2019) argue that empowering leadership can enhance innovative work-behavior, particularly through increased knowledge sharing. Knowledge sharing may be especially important during the COVID-19 pandemic to support decision making in organizations (Lee Y. et al., 2020).

Based on the account above, it can be argued that empowering leadership will grant employees more decision power and job autonomy (Lorinkova et al., 2013), show them what their work means to strategic direction of their organization (Ahearne et al., 2005; Martin et al., 2013) and express confidence in their own abilities which will drive them to accept more responsibility (Cheong et al., 2019). This enhances their motivation and perceived opportunities to enact innovative work-behavior, also when working from home. Hence, we propose the following hypothesis:

*H1: Empowering leadership has a positive direct relationship with innovative work-behavior.*

### Directive Leadership and Innovative Work-Behavior

Directive leaders aim to reduce ambiguity and increase process efficiency by structuring the work of their employees and providing them with clear goals (Pearce and Sims, 2002; Lorinkova et al., 2013). Conversely to empowering leadership, directive leaders attempt to maximize the performance of their employees by centralizing decision power (Stoker et al., 2019). This type of leadership may be beneficial in times of crisis to manage uncertainty and avoid loss of performance (Yun et al., 2005; Stoker et al., 2019). In relation to innovative work-behavior, however, directive leadership might prove to be rather detrimental (Somech, 2005). Innovative work-behavior is fueled through leader-member exchange and when employees experience greater decision latitude and autonomy, this will benefit their innovative work-behavior (Scott and Bruce, 1994). Directive leadership might negatively relate to these attributes (Stoker et al., 2019) and, therefore, might provide an environment in which employees struggle to be innovative due to a lack of freedom to explore and bring forth new ideas. Therefore, we propose the following hypothesis:

*H2: Directive leadership has a negative direct relationship with innovative work-behavior.*

### The Mediating Role of Worked-Related Flow in the Relationship Between Leadership and Innovative Work-Behavior

While flow might be experienced through a wide array of activities (Csikszentmihalyi, 1975), Bakker (2008) examined flow in the context of work activities and presented a three-dimensional conceptualization of work-related flow: pleasure, intrinsic motivation, and absorption. Generally, flow seems to be beneficial for creativity and innovative behaviors, since intrinsically motivated persons tend to be learning oriented, cognitively flexible, and willing to take risks (Amabile et al., 2005). This is echoed by the work of Maqbool et al. (2018) who argue that work-related flow can enhance innovative work-behavior, as employees benefit from the increased intrinsic motivation, enjoyment, and absorption in their ability to create and promote new ideas. Another benefit is that higher degrees of work-related flow can lead to higher energy and allow employees to recover energy quicker (Demerouti et al., 2011). This might be exceptionally relevant in the current situation, as the restrictions imposed in response to the COVID-19 pandemic could impede employees’ energy levels and, therefore, their innovative work-behavior (Montani and Staglianò, 2021).

Aside from the direct effect of work-related flow on innovative work-behavior, leadership may also relate to innovative work-behavior indirectly via work-related flow. Empowering leadership, for instance, can increase employees’ intrinsic work-motivation, one of the dimensions of flow, and creativity (Bakker, 2008; Zhang and Bartol, 2010). A study by Peters et al. (2014) shows that when employees feel empowered (experience more job autonomy, work from home and experience coaching leadership), they experience more work-related flow. From a self-determination perspective, Hon and Chan (2013) demonstrate that empowering leaders can foster intrinsic motivation, one of the dimensions of work-related flow (Bakker, 2008), among followers, resulting in their ability to create novel ideas being enhanced (Zhang and Bartol, 2010). Higher flow levels could positively relate to the degree to which employees shape their role to their own competencies and preferences (Bakker and Van Woerkom, 2017). Based on these arguments, we propose the following hypothesis:

*H3a: Work-related flow (partly) positively mediates the positive relationship between empowering leadership and innovative work-behavior.*

Directive leadership, on the other hand, may decrease the degree of work-related flow, as it removes autonomy through issuing instructions (Stoker et al., 2019). While directive leadership may support work-related flow by providing clear goals (Quinn, 2005), the issuing of instructions on how to approach one’s work (Pearce and Sims, 2002) may decrease employees’ autonomy and, hence, intrinsic motivation to seek new innovative solutions in their work (Scott and Bruce, 1994) and decrease their work engagement during homeworking (Sardeshmukh et al., 2012). Similarly, directive leadership can limit employees’ opportunities in their work to create a better job-fit, thereby risking lower degrees of work-related flow (Bakker and Van Woerkom, 2017). While acknowledging the evidence of a potential positive effect of directive leadership on work-related flow, we argue that this leadership style negatively correlates to employees’ ability and motivation to be innovative. Therefore, we propose the following:

*H3b: Work-related flow (partly) negatively mediates the negative relationship between directive leadership and innovative work-behavior.*

### The Moderating Role of IT-Enabled Presence Awareness in the Relationship Between Leadership and Work-Related Flow

While the concept of remote (home) working is not new, it has seen a tremendous growth over the past year, resulting from the lockdown measures (Contreras et al., 2020). This increase has implications for how concepts such as leadership (Contreras et al., 2020), work-related flow (Peters et al., 2014), manifest themselves and are interrelated. In a remote work context, both employees and their leaders need to be accessible online to interact (Malhotra and Majchrzak’s, 2014). IT-enabled presence awareness gives employees the sense that their leaders and their teams are accessible and available to engage and collaborate with (Malhotra and Majchrzak’s, 2014; Lim, 2018).

When employees experience high degrees of IT-enabled presence awareness, they perceive their empowering leaders to be accessible and reachable through the provided technical channels (Lim, 2018). In this case, they will experience higher degrees of access to their leaders’ encouragement and support while being able to rapidly ask for feedback and clarification on instructions they receive, which may enhance their work-related flow (Peters et al., 2014; Lim, 2018; Wang and Shaheryar, 2020). Whilst empowering leadership can enhance employees’ work-related flow in remote work contexts (Peters et al., 2014), once employees experience low IT-enabled presence awareness, they may feel a diminished accessibility to the encouragement and support of their leaders (Malhotra and Majchrzak’s, 2014; Lim, 2018). Subsequently, they may experience lower absorption and motivation in their work (Peters et al., 2014). After all, prior studies on the relationship between empowerment of employees and their work-related flow have emphasized the importance of the relationship between the leader and the employee, as being one of mutual trust (Peters et al., 2014; Wang and Shaheryar, 2020).

Directive leadership entails leaders providing employees with goals, instructions on how to approach these goals and reprimand when work is not up to par (Pearce et al., 2003). When employees experience high degrees of IT-enabled presence awareness, they perceive their leaders to be accessible and reachable through the provided technical channels (Lim, 2018). In this case, they will experience higher degrees of access to their leaders’ encouragement and support while being able to rapidly ask for feedback and clarification on instructions they receive, which may enhance their work-related flow (Peters et al., 2014; Lim, 2018; Wang and Shaheryar, 2020). However, when employees feel that their opportunity to ask for clarification on given instructions and feedback on their work is diminished due to low IT-enabled presence awareness they may experience a higher risk for misinterpretations that may result in reprimand or damage the trust that leaders place in them (Malhotra and Majchrzak’s, 2014; Lim, 2018). Therefore, they may experience less work-related flow (Peters et al., 2014). Hence, we conjecture the following moderation hypotheses:

*H4a: Perceived IT-enabled presence awareness will positively moderate the direct relationship between empowering leadership and work-related flow, such that this relationship will be stronger for employees who perceive higher levels of IT-enabled presence awareness than for employees who perceive lower degrees of IT-enabled presence awareness.*

*H4b: Perceived IT-enabled presence awareness will negatively moderate the direct relationship between directive leadership and work-related flow, such that this relationship will be weaker for employees who perceive higher levels of IT-enabled presence awareness than for employees who perceive lower degrees of IT-enabled presence awareness.*

### The Relation of Time to the Relationships Between Leadership, Work-Related Flow, IT-Related Presence Awareness and Innovative Work-Behavior

According to Bandura (1995), “innovations demand heavy investment of effort over a long period with uncertain results” (p. 13). Hence, it is important to consider the implications of leadership on innovative work-behavior over multiple moments in time (Lorinkova et al., 2013; Lee A. et al., 2020). The demand for more sophisticated research designs becomes especially prominent in the light of the COVID-19 pandemic, as influences of leadership choices in response to the crisis (Stoker et al., 2019) are being unveiled at this very moment (Bajaba et al., 2021). The benefits of empowering leadership and directive leadership for employee performance may especially become clear in the long-term, depending on the employees’ growth in their competencies and responsibility (Lorinkova et al., 2013).

The direct relationship of empowering leadership with innovative work-behavior does not only stem from more autonomy, trust and involvement, but also from leaders who are sharing knowledge, meaningfulness and providing confidence in the employees’ capabilities to be innovative in the light new work situation (Ahearne et al., 2005; Martin et al., 2013; Montani and Staglianò, 2021). These latter two attributes of empowering leadership might have been especially important for innovative work-behavior in the earlier phases of the COVID-19 pandemic when employees might have been more unsure how to respond to the new work situation and rely more on their leaders’ knowledge and support (Carnevale and Hatak, 2020). However, as time progressed and the COVID-19-measures are prolonged and intensified, employees perceiving empowering leadership might become more self-confident and proactive (Coun et al., 2021) in the new way of working and their role and responsibilities toward innovation (Lorinkova et al., 2013; Cheong et al., 2019). Hence, while they may continue to experience empowering leadership, they may rely less on their leaders’ support in providing meaningfulness to their work in relation to the (new) organizational direction (Ahearne et al., 2005) to display adequate innovative work-behavior in comparison to the earlier phases of the pandemic.

At the same time, the indirect relationship between empowering leadership and innovative work-behavior via work-related flow may have become stronger as employees have grown more competent and confident with taking responsibilities in the light of new strategic goals, perhaps as a result of longer exposure to empowering leadership while working from home (Lorinkova et al., 2013; Cheong et al., 2019). In a similar vein, employees that experience empowering leadership in the second phase of the COVID-pandemic may also have had the time to shape their role to better fit with their intrinsic motivation, enhancing their work-related flow (Bakker and Van Woerkom, 2017). This may imply that they will feel more able and motivated to take the opportunity to pursue challenges, such as displaying innovative work-behavior (Scott and Bruce, 1994; Bandura, 1995). Based on this account, the following hypotheses were developed:

*H5a: The strength of the positive direct relationship between empowering leadership and innovative work-behavior will be weaker across time (T2 in comparison with T1).*

*H5b: The strength of the positive indirect relationship between empowering leadership and innovative work-behavior via work-related flow will be stronger across time (T2 in comparison with T1).*

In the case of directive leadership, employees are granted less autonomy and responsibility and do have less insight into the strategic direction of the organization in comparison to empowering leadership. Therefore, when subject to higher degrees of directive leadership, employees may engage less in task learning and may develop less psychological empowerment and confidence in their competencies (Lorinkova et al., 2013). As the relationship between leader and employee is important for the development of competencies that support innovative work-behavior (Scott and Bruce, 1994), employees’ innovative work-behavior during the COVID-19 pandemic could be increasingly negatively directly influenced by directive leadership as time passes.

In a similar vein, employees may also experience lower levels of work-related flow due to having had less opportunity to craft their job to their competencies and intrinsic interests (Bakker and Van Woerkom, 2017), therefore, creating an increasingly unfavorable environment for employees’ innovative work-behavior (Scott and Bruce, 1994; Zhang and Bartol, 2010). This would hint at a stronger indirect relationship between directive leadership and innovative-work-behavior via work-related flow.

*H5c: The strength of the negative direct relationship between directive leadership and innovative work-behavior will be stronger across time (T2 in comparison with T1).*

*H5d: The strength of the negative indirect relationship between directive leadership and innovative work-behavior via work-related flow will be stronger across time (T2 in comparison with T1).*

While the restrictions to avoid infection with the coronavirus decreased the communication richness within the workforce (Martins et al., 2004; Garro-Abarca et al., 2021), employees who work from home and are not co-located may adapt to the new technology and ways of working as time passes (Majchrzak et al., 2000). According to Axtell et al. (2004), relations between employees working together through technology-enabled communication could adapt and be equal to face-to-face if granted enough time. A study conducted on a new virtual team that worked on creating an innovative product (Majchrzak et al., 2000), showed that after an initial period of misalignments, employees adapted to the use of technology succeeded in their work. When employees become more familiarized with the use of new technologies and collaborating from different locations over time, it could be argued that they will rely less on their leaders’ behaviors to guide them in their work-related flow. According to Bakker and Van Woerkom (2017), employees may enhance work-related flow through self-determination strategies that are supported by their leaders. Based on these arguments, we expect that employees adapt to the new ways of IT-enabled working over a prolonged time working from home in the pandemic, therefore, weakening the potential influence of IT-enabled presence awareness on the relationships between the leadership styles and work-related flow.

*H5e: The strength of the moderating influence of IT-enabled presence awareness on the direct relationship between empowering leadership and work-related flow will be weaker across time (T2 in comparison with T1).*

*H5f: The strength of the moderating influence of IT-enabled presence awareness on the direct relationship between directive leadership and work-related flow will be weaker across time (T2 in comparison with T1).*

**FIGURE 1:**
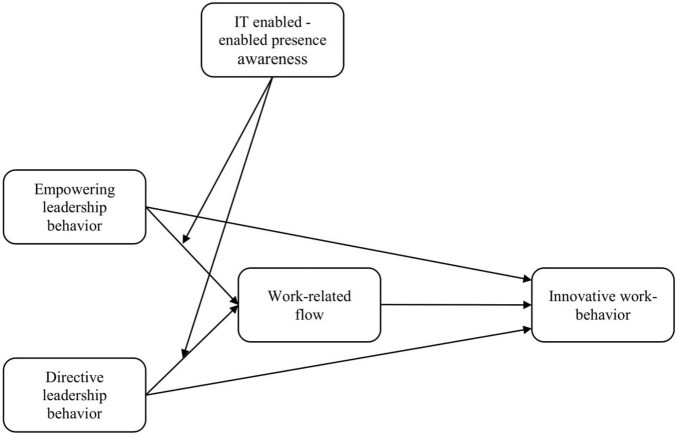
Hypothesized relationships in the conceptual model.

Figure 1 depicts the hypothesized relationships in the conceptual model.

## Materials and Methods

### Sample

Data was gathered by utilizing an online questionnaire, aimed to derive perceived experiences of the respondents. The original sample consisted of 377 respondents who were invited to fill out a survey in the period July–August 2021 which represented the First Wave (T1) and who were asked to complete the same survey in the period November-December 2021 which represented the Second Wave (T2). Out of these 377 only 257 respondents completed the survey completely during the second wave. Hence, these 257 respondents were used to compare the First Wave and the Second Wave. The surveys were collected using a personal network and a virtual work consultancy bureau. A descriptive analysis was conducted to show a more sophisticated view on the sample, which is depicted in Table 1. Also, a *T*-test was conducted to see whether the actual working hours at home on a weekly basis differed in the First Wave in comparison with the Second Wave. A significant difference was found to be lower between actual working hours at home in the First Wave (*M* = 30.23; *SD* = 9.19) and the actual working hours at home in the Second Wave (*M* = 33.66; *SD* = 8.60). This difference was significant (*t*(246) = 51.67, *p* < 0.000), indicating that during the Second Wave more hours (3.63) were worked at home. As depicted in Tables 1–3, some respondents didn’t fill out all items of the survey. After a first inspection we replaced all missing data points with the mean value of all remaining data points per column (i.e., indicator or variable). We chose for this as mean replacement has the benefit not to alter the sample size and the mean value of variables in the sample (cf. Hair et al., 2014).

**TABLE 1:** Descriptive overview of the sample.

			**Innovative work-behavior**	
	***N***	**%**	**Mean**	***SD***
**Gender**				
Male	116	45.14%	3.29	0.62
Female	131	50.97%	3.15	0.61
Unknown/missing	10	3.89%		
**Age categories**				
<31	14	5.45%	3.46	0.59
31–40	49	19.07%	3.15	0.65
41–60	161	62.65%	3.22	0.6
above 60	21	8.17%	3.29	0.55
Unknown/missing	12	4.66%		
**Domestic situation**				
Live-in partner/no live-in children	71	27.63%	3.18	0.55
Live-in partner/live-in children	121	47.08%	3.23	0.6
Single parent/live-in children	18	7.00%	3.45	0.62
Single parent/no live-in children	35	13.62%	3.18	0.75
Unknown/missing	12	4.67%		
**Relation**				
Yes	192	74.71%	3.27	0.71
No	53	20.62%	3.21	0.58
Unknown/missing	12	4.67%		
**Industry**				
Municipal City	55	21.40%	3.37	0.67
Government	120	46.69%	3.08	0.54
Food Industry	22	8.56%	3.24	0.56
University	23	8.95%	3.27	0.76
Housing cooperative	19	7.39%	3.43	0.69
Other industries	18	7.01%	3.56	0.53

**TABLE 2:** Construct descriptive statistics First Wave.

**First Wave**	***N***	**Theoretical range**	**Actual range**	**Mean**	***SD***	**Cronbach’s Alfa**	**AVE**
Empowering leadership	244	1.00–5.00	1.13–5.00	3.64	0.65	0.87	0.52
Directive leadership	247	1.00–5.00	1.00–5.00	3.1	0.74	0.72	0.63
IT-enabled presence awareness	256	1.00–5.00	1.00–5.00	3.26	0.92	0.78	0.65
Work-related flow	248	1.00–7.00	1.36–6.55	4.32	0.87	0.89	0.5
Innovative work- behavior	249	1.00–5.00	1.33–5.00	3.22	0.62	0.92	0.6

**TABLE 3:** Construct descriptive statistics Second Wave.

**Second Wave**	***N***	**Theoretical range**	**Actual range**	**Mean**	***SD***	**Cronbach’s Alfa**	**AVE**
Empowering leadership	234	1.00–5.00	1.13–5.00	3.55	0.72	0.90	0.57
Directive leadership	235	1.00–5.00	1.00–5.00	3.12	0.72	0.70	0.59
IT-enabled presence awareness	256	1.00–5.00	1.00–5.00	3.19	0.88	0.70	0.68
Work-related flow	243	1.00–7.00	1.82–6.55	4.20	0.89	0.91	0.61
Innovative work- behavior	246	1.00–5.00	1.33–5.00	3.21	0.63	0.92	0.61

### Measures

All constructs in the proposed model were based on reflective multi-item scales. The instruments used consisted of measures for the research constructs as described in this section. As the interest within this research lies in measuring the general influence between these constructs, we used the combined subscales from which they are composed.

#### Empowering Leadership

Empowering leadership was measured using the validated questionnaire of Ahearne et al. (2005). The questionnaire is comprised out of four multi-item subscales (enhancing the meaningfulness of work, fostering participation in decision making, expressing confidence in high performance and providing autonomy). A five-point Likert’s scale, where 1 represented “strongly disagree” and 5 represented “strongly agree” was used. An example item we used is the following: “My manager helps me understand how my objectives and goals relate to that of the company”.

#### Directive Leadership

Directive leadership was measured using Pearce et al. (2003) dimensions for directive leadership behavior: assign goals, instruction and command, and reprimand. We used a shorter version of the scale which included one item for each of the subscales. Items for this construct were measured through a five-point Likert’s scale, where 1 represented “strongly disagree” and 5 represented “strongly agree.” An example item included is: “My team leader gives me instructions about how to do my work”.

#### Work-Related Flow

Work-related flow was measured according to the scales developed by Bakker (2008), which constituted absorption, intrinsic motivation and work enjoyment. In line with the study of Bakker (2008) we used a seven-point Likert’s scale, where 1 represented “never” and 7 represented “always.” An example item for this construct is: “I work because I enjoy it”.

We measured *IT-enabled presence awareness* by using the three-item measure as described by Malhotra and Majchrzak’s (2012, 2014). We used a five-point Likert’s scale, where 1 represented “strongly disagree” and 5 represented “strongly agree.” An example item for this measurement is: “The digital technology makes me feel as if I am present in the same location as my colleagues (even when they are not)”.

To measure individual *innovative work-behavior* we used Janssen’s (2000) validated questionnaire. The three scales (idea generation, idea promotion and idea realization) that constitute the questionnaire include nine items in total. We used a five-point Likert’s scale, where 1 represented “never” and 5 represented “always” through which respondents indicated how often they experienced the statements. An example item we used for idea generation is: “I create new ideas for difficult issues”.

### Procedure

Preparation tests were conducted using SPSS version 27, to ensure the data was sufficiently prepared before the actual analysis. Descriptive and frequency analyses were conducted to gain a better perspective about the characteristics of the sample.

This research utilizes a PLS-SEM analysis (version 3.3.3 Smart PLS) to check the validity, reliability and factor loading of the data (Ringle et al., 2015). Although, the sample of 257 respondents was shown to be normally distributed, a bootstrapping method in PLS-SEM was utilized to increase the predictive power of the sample (Hair et al., 2014).

## Results

### Model Characteristics

First, the reliability of the outer model for each of the waves were examined. As shown in Tables 2, 3, the reliability scores were all deemed acceptable. The scales for all the constructs are shown to be reliable in terms of indicator validity since the Cronbach Alphas passed the threshold value of 0.70 as given by Hair et al. (2014). After verifying the composite validity of the constructs, they were checked for convergent validity.

In order to have enough convergent validity the Average Variance Extracted (AVE) needs to exceed the value of 0.50 (Fornell and Larcker, 1981). As, empowering leadership (AVE: 0.42), work-related flow (AVE: 0.48) and IT-enabled presence awareness (AVE: 0.40) demonstrated insufficient convergent validity according to the Fornell and Larcker criterion, we increased convergent validity by deleting items. The items with the least factor loadings were removed first with checking whether the remaining items still provided a proper representation of the overall construct. Analyses with the PLS-algorithm were step by step repeated to increase sufficient reliability, convergent validity and discriminant validity (cf. Ringle et al., 2015). Henceforth, one item (item 7) was deleted of the empowering leadership variable to have adequate reliability and convergent validity. Furthermore, two items (items 1 and 3) were deleted of the work-related flow variable. Finally, one item (item 1) was deleted of the IT-enabled presence awareness variable. No items were deleted of the innovative work-behavior and directive leadership variable as these variables demonstrated enough reliability and convergent validity.

The final examination is focused on assessing the discriminant validity of the constructs for each of the two waves, by examining and comparing the AVEs of each respective construct with the inter-construct correlations in the model. Thereby, determining for each latent variable shared greater variance with its own measurement items or with the other constructs (Fornell and Larcker, 1981; Chin, 1998). When comparing the square roots of the AVE’s for each respective construct with the correlations between the constructs in the model, it can be seen in Tables 4, 5 that none of the correlations exceeds the value of the square root of the AVE. Therefore, it can be concluded that all constructs can be considered sufficient in terms of both reliability and validity.

**TABLE 4:** Correlations first wave and the square root of the Average Variance Extracted (in bold).

**First wave**	**Empowering leadership**	**Directive leadership**	**IT-enabled presence awareness**	**Work-related flow**	**Innovative work-behavior**
Empowering leadership	**0.72**				
Directive leadership	0.27**	**0.79**			
IT-enabled presence awareness	0.11*	0.09**	**0.81**		
Work-related flow	0.33**	–0.02	0.05	**0.71**	
Innovative work- behavior	0.20**	−0.19**	0.08	0.33**	**0.78**

Significance correlations: **p < 0.01, *p < 0.05.

**TABLE 5:** Correlations second wave and the square root of the Average Variance Extracted (in bold).

**Second wave**	**Empowering leadership**	**Directive leadership**	**IT-enabled presence awareness**	**Work-related flow**	**Innovative work-behavior**
Empowering leadership	**0.75**				
Directive leadership	0.33**	**0.77**			
IT-enabled presence awareness	0.06	0.12**	**0.83**		
Work-related flow	0.39**	–0.070	0.12*	**0.73**	
Innovative work- behavior	0.14*	−0.23**	0.08	0.38**	**0.78**

Significance correlations: **p < 0.01, *p < 0.05.

### Model Estimations

This subsection covers the inner model evaluation and estimates for each wave. Bootstrap t-statistics were used for testing the significance of the path-coefficients (Anderson and Gerbing, 1988). This bootstrapping was performed with 5000 subsamples, with a bias-corrected bootstrap, utilizing a 95% significance at a two-tailed test. First, an estimation of the direct effects of directive leadership and empowering leadership directly and via work related flow on innovation showed that the model for the First Wave explained a variance (*R*^2^) of 0.17 for innovative work-behavior and a variance (*R*^2^) of 0.14 for work-related flow. For the Second Wave innovative work-behavior demonstrated a variance (*R*^2^) of 0.19 and work-related flow a variance (*R*^2^) of 0.23. Furthermore, an estimation of model fit was made with a Standardized Root Mean Square Residual (SRMR) which is the commonly used model fit indicator in PLS-SEM analysis (cf. Hair et al., 2014; Ringle et al., 2015), showing a value of 0.08 for the First Wave and for the Second Wave separately which are in accordance with the criterion set by Hu and Bentler (1998). Henceforth, the model displayed good model fit. We used the results of the First Wave to test hypotheses 1, 2, 3a, and 3b.

Second, to test the mediation effect of work-related flow in the model, we calculated the indirect effects of directive leadership and empowering leadership via work-related flow on innovative work-behavior using the PLS-SEM algorithm (Hair et al., 2014). Lastly, we examined the moderation effect of the construct IT-enabled presence awareness on the relation between empowering leadership and work-related flow, and on the relation between directive leadership and work-related flow using the PLS-SEM algorithm as well. In the Tables 6, 7 the results are depicted.

**TABLE 6:** Structural direct relationships with path coefficients (γ) for the first wave (T1) and second wave (T2).

**First wave vs. Second wave**	**Coefficient (γ) Wave 1**	**Coefficient (γ) wave 2**	**SD Wave 1**	**SD Wave 2**	***P*-Value Wave 1**	***P*-Value Wave 2**	**Hypotheses**
Empowering leadership –>Innovative work-behavior	0.18	0.09	0.08	0.08	0.02	0.23	H1, H5a
Empowering leadership –>Work-related flow	0.35	0.48	0.06	0.06	0.00	0.00	H4a
Empowering leadership x IT-enabled presence awareness –>Work-related flow	0.05	0.05	0.07	0.07	0.46	0.50	H4a, H5e
Directive leadership –>Innovative work-behavior	−0.23	−0.24	0.09	0.06	0.01	0.00	H2, H5c
Directive leadership –>Work-related flow	−0.12	−0.25	0.12	0.07	0.33	0.00	H4b
Directive leadership x IT-enabled presence awareness –>Work-related flow	0.09	0.13	0.09	0.08	0.28	0.09	H4b, H5f

**TABLE 7:** Structural indirect relationships with path coefficients (γ) for the first wave (T1) and second wave (T2).

**First wave vs. Second wave**	**Coefficient (γ) Wave 1**	**Coefficient (γ) Wave 2**	**SD Wave 1**	**SD Wave 2**	***P*-Value Wave 1**	***P*-Value Wave 2**	**Hypotheses**
Empowering leadership –>Work-related flow –>Innovative work-behavior	0.10	0.16	0.03	0.04	0.00	0.00	H3a, H5b
Directive leadership –>Work-related flow –>Innovative work-behavior	−0.03	−0.08	0.04	0.03	0.39	0.01	H3b, H5d

*Hypothesis 1*

Hypothesis one is supported as a positive relationship was found between empowering leadership and innovative work-behavior and was furthermore shown to be significant (γ = 0.18, *p* < 0.05, *R*^2^ = 0.17).

*Hypothesis 2*

The second hypothesis, which suggests a negative relationship between directive leadership and innovative work-behavior, demonstrated to be negative and significant. Therefore, the hypothesis is supported by the analysis (γ = −0.23, *p* < 0.01, *R*^2^ = 0.17).

*Hypothesis 3a and 3b*

Hypotheses 3a and 3b suggested a mediating effect of work-related flow on the relationship between empowering leadership and innovative work-behavior (H3a) and on the relationship between directive leadership and innovative work-behavior (H3b). H3a was supported as the indirect effect of empowering leadership on innovative work-behavior via work-related flow demonstrated to be significant (γ = 0.10, *p* < 0.00, *R*^2^ = 0.17). H3b was not supported as the indirect effect of directive leadership on innovative work-behavior via work-related flow demonstrated to be not significant (γ = −0.03, *p* = 0.39, *R*^2^ = 0.17).

*Hypothesis 4a and 4b*

The fourth hypotheses suggested a moderating effect of IT-enabled presence awareness as well on the relationship between empowering leadership and work-related flow as on the relationship between directive leadership and work-related flow. H4a was not supported as the moderating effect of IT-enabled presence awareness on the relation between empowering leadership on work related flow demonstrated to be not significant (γ = 0.05, *p* = 0.46, *R*^2^ = 0.14). H4b was also not supported as the moderating effect of IT-enabled presence awareness on the relation between directive leadership on work-related flow demonstrated to be not significant (γ = 0.09, *p* = 0.28, *R*^2^ = 0.14).

**FIGURE 2:**
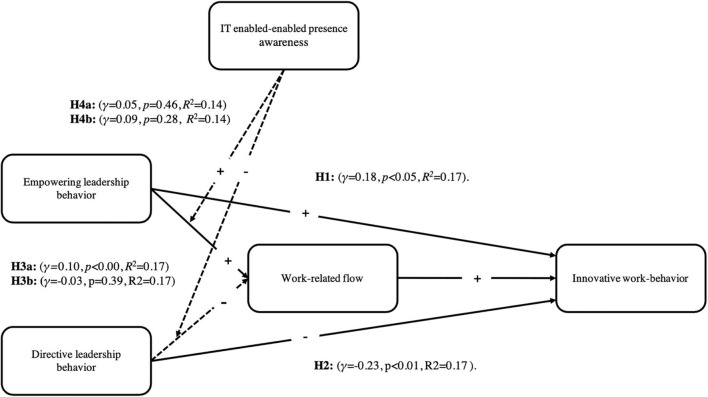
Significant and non-significant relationships of the conceptual model in the first wave.

Figure 2 depicts the conceptual model of the first wave. Full arrows indicate significant relationships while dotted arrows indicate not significant relationships.

*Hypotheses 5a, 5b, 5c, 5d, 5e, and 5f*

Hypothesis 5 encompasses six hypotheses which focuses on the specific time related effects on the direct and indirect relations in the model looking at the differences in the coefficient (γ) and its significance between the First Wave (T1) and the Second Wave (T2). For H5a support was found as the relationship between empowering leadership and innovative work-behavior was not significant in the Second Wave (γ = 0.09, *p* = 0.23, *R*^2^ = 0.19), while this relationship was significant in the First Wave (γ = 0.18, *p* < 0.05, *R*^2^ = 0,17). For H5b support was found as the coefficient (γ = 0.16, *p* < 0.00, *R*^2^ = 0.19) of the indirect relationship between empowering leadership and innovative work-behavior via work related flow increased in comparison with the First Wave (γ = 0.10, *p* < 0.00, *R*^2^ = 0.17). For H5c support was found as the relationship between directive leadership and innovative work-behavior became more significant in the Second Wave (γ = −0.24, *p* < 0.00, *R*^2^ = 0,19) in comparison to the First Wave (γ = −0.23, *p* < 0.01, *R*^2^ = 0.17). For H5d support was found as the indirect relationship between directive leadership and innovative work-behavior via work related flow demonstrated to become significant (γ = −0.08, *p* < 0.01, *R*^2^ = 0.19) while this relation showed to be not significant in the First Wave (γ = −0.03, *p* = 0.39, *R*^2^ = 0.17). H5e was not supported as also the moderating effect of IT-enabled presence awareness on the relationship between empowering leadership and work-related flow demonstrated not to be significant (γ = 0.05, *p* = 0.50, *R*^2^ = 0.23) in the Second Wave, just as in the First Wave (γ = 0.05, *p* = 0.46, *R*^2^ = 0.14). H5f was not supported as also the moderating effect of IT-enabled presence awareness on the relationship between directive leadership and work-related flow demonstrated not to be significant (γ = 0.13, *p* = 0.09, *R*^2^ = 0.23) in the Second Wave, just as in the First Wave (γ = 0.09, *p* = 0.28, *R*^2^ = 0,14).

**FIGURE 3:**
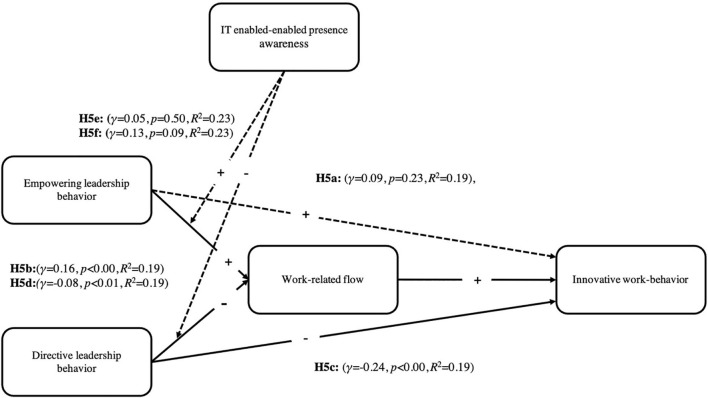
Significant and non-significant relationships of the conceptual model in the second wave.

Figure 3 depicts the conceptual model of the second wave. Full arrows indicate significant relationships while dotted arrows indicate not significant relationships.

## Discussion

Employing a data set of 257 employees, the present study aimed to contribute to the conversation on leadership and innovation by examining the mediating role of work-related flow between empowering and directive leadership and innovative work-behavior in times of COVID-19 and the moderating role of IT-enabled presence awareness in these relationships. Furthermore, we examined how these relationships differ across time. Below, we will discuss the study’s main findings and contributions in the light of theory. We conclude by discussing its limitations and implications for research and management practice.

### The Relationship Between Empowering and Directive Leadership on Innovative Work-Behavior

In line with expectations, we found that empowering leadership during the earlier phases of the COVID-19 pandemic was positively related to innovative work-behavior. This finding corroborates with previous empirical studies on leadership and innovative behavior (Scott and Bruce, 1994; Zhang and Bartol, 2010). The COVID-19 pandemic challenged leaders to create an engaging work environment where employees feel supported and are enabled to perform to the best of their abilities, despite their inability to meet with them face-to face (Contreras et al., 2020). Empowering leadership grants employees with more latitude to approach their work and encourages participation in decision making, which expresses confidence in the employees’ abilities (Martin et al., 2013). Moreover, empowering leadership provides guidance in the strategic direction of the goals of the entire organization and the meaningfulness of the employees’ work in these goals (Ahearne et al., 2005). This supports employees directly in their individual innovative work-behavior (Scott and Bruce, 1994; Amabile and Pratt, 2016) and might be especially important in the first phase of the pandemic, when employees might have been less sure on how to respond (Montani and Staglianò, 2021).

In line with our expectations, we found that directive leadership during the initial phases of the COVID-19 pandemic negatively influenced innovative work-behavior. This finding chimes with the work of Somech (2005), who argued that directive leadership has a potential detrimental effect on employees’ innovative behavior. While directive leadership was shown to benefit initial task performance by focusing employees’ attention on executing their specific tasks (Lorinkova et al., 2013), this can be different in case of innovative behavior where the need for autonomy to explore new ideas and solutions is imperative (Zhang and Bartol, 2010). Based on our findings, it can be argued that the lack of decision latitude and autonomy that employees may experience as a result of directive leadership (Stoker et al., 2019) can have hampered their innovative work-behavior as they experienced scarce opportunity to explore new ideas (Scott and Bruce, 1994).

Our findings regarding the relationships of empowering and directive leadership with innovative behavior during the initial phases of the COVID-19 pandemic extend the leadership literature (Lorinkova et al., 2013; Stoker et al., 2019; Contreras et al., 2020; Bajaba et al., 2021) by providing insights into how both leadership styles influence employees’ behavior during a crisis, such as the COVID-19 pandemic, which required intensive remote homeworking. During the initial phases of a crisis, some leaders might have shown a proclivity to become more directive to reduce ambiguity (Stoker et al., 2019). However, our study suggests that in the early phase of the pandemic, empowering employees by emphasizing the meaningfulness of their work in relation to the organization’s strategic direction, expressing confidence by broadening their responsibilities and decision power, and giving them more freedom to explore novel ideas (Ahearne et al., 2005; Martin et al., 2013) fostered more idea generation, promotion, and realization (Janssen, 2000).

### The Mediating Role of Worked-Related Flow in the Relationship Between Leadership and Innovative Work-Behavior

In line with expectations, we found that the relationship between empowering leadership and innovative work-behavior during the initial phases of the COVID-19 pandemic was partly mediated through work-related flow. This outcome chimes with a study by Peters et al. (2014) who argued that when homeworkers are empowered, they experience more work-related flow. Subsequently, work-related flow can result in more autonomous motivation and absorption in work, which enhances creativity and innovative work-behavior (Hon and Chan, 2013; Maqbool et al., 2018). Our study underlines the importance of work-related flow as a (partial) mediator between empowering leadership and innovative behavior amongst employees who are intensively working from home.

Contrary to our expectations, however, we did not find significant evidence for the proposed negative indirect relationship between directive leadership and innovative work-behavior via work-related flow during our First Wave of data collection. A possible explanation for this outcome could be that even though directive leaders may have aimed to influence the performance of employees (Lorinkova et al., 2013), this did not enhance employees’ absorption, enjoyment, and motivation during work (Bakker, 2008). Hence, leaders that initially focused on reducing ambiguity through issuing commands and assigning goals as a response to the changing working conditions during the COVID-19 pandemic (Stoker et al., 2019) seem to have been less successful in influencing employees’ intrinsic abilities and mental state through work-related flow (Bakker, 2008).

### The Moderating Role of IT-Enabled Presence Awareness in the Relationship Between Leadership and Work-Related Flow

Our analyses did not provide evidence for the proposed moderating role of IT-enabled presence awareness on the relationship between leadership and work-related flow during the initial phases of the COVID-19 pandemic. A possible explanation for this outcome could be that presence awareness among remote workers could be influenced by characteristics of the existing relationship with their co-workers (Gruenfeld et al., 1996; Malhotra and Majchrzak’s, 2014; Lim, 2018) and other contextual factors (Lim, 2018). For example, when employees have been acquainted before the COVID-19 pandemic and, therefore, must cross few so-called ‘knowledge boundaries,’ their shared context might already provide presence awareness (Hinds and Mortensen, 2005). In that case, the degree of IT-enabled presence awareness does not impact the relationship between the perceived leadership style and work-related flow. In a similar vein, Lim (2018) acknowledges that some aspects of leadership behavior may be suitable to be conveyed via asynchronous information technology (such as email) and not necessarily via the pathway of IT-enabled presence awareness. Hence, there might have been factors that influenced the experience of employees experienced presence awareness, which we did not include in our research and explain the not significant result.

### The Relation of Time to the Relationships Between Leadership, Work-Related Flow, and Innovative Work-Behavior

Besides providing insight into the mechanisms that influence innovative work-behavior during the initial phases of the COVID-19 pandemic, we also found significant evidence of temporal effects as the relationships between leadership, work-related flow and innovative work-behavior may differ across different phases.

Regarding empowering leadership, we did find a weaker direct relationship with innovative work-behavior during the second phase compared to the first phase, however, the relationship was not significant Contrarily, the indirect relationship between these two constructs via work-related flow was stronger in the second phase compared with the first phase. These outcomes indicate that as time passes, empowering leadership remains important, but relates to innovative behavior increasingly via work-related flow as an underlying mechanism. This could be explained by employees feeling more comfortable with the new situation, their organization’s response to the changing environment and their work, than during the earlier phase in the pandemic (Montani and Staglianò, 2021). Therefore, they may rely less on the guidance of their leader in meaningfulness of their work and encouragement to partake in decision making (Ahearne et al., 2005). Instead, they draw more upon the autonomy and intrinsic motivation provided by empowering leadership to fuel their work-related flow. These results corroborate with Lorinkova et al. (2013) who argue that the positive effects of empowering leadership over time lie in that it facilitates the development of employees’ competencies and build confidence in their own abilities to take of broader responsibilities as a result of being empowered (Cheong et al., 2019). As the new ways of working resulting from the COVID-19 pandemic was prolonged, employees who receive emotional support, encouragement (Hon and Chan, 2013) and freedom to shape their work situation to their own preferences (Bakker and Van Woerkom, 2017) also experience more work-related flow which enhanced their innovative work-behavior (Scott and Bruce, 1994).

Interestingly, in the second phase of the COVID-19 lockdown which intensified homeworking, we observed (more) negative relationships between directive leadership and innovative work-behavior, both directly and indirectly, via work-related flow. The direct relationship was stronger both in terms of strength and significance. This may indicate that when the leadership style does not provide employees with greater decision latitude and autonomy, this will negatively affect employees’ innovative work-behavior (Scott and Bruce, 1994). In a similar vein, our study indicates a negative relationship between directive leadership and work-related flow and, subsequently, innovative work-behavior. Possibly, employees experiencing directive leadership during the COVID-19 pandemic received little support and opportunity to develop their competencies (Lorinkova et al., 2013). In other words, directive leaders that likely prefer issuing commands without much input from the employees themselves might not provide employees with enough autonomy for them to experience engagement in their work-activities (Bakker and Van Woerkom, 2017; Stoker et al., 2019). Consequently, employees are less likely to experience work-related flow (Bakker, 2008; Bakker and Van Woerkom, 2017), which can come at the expense of their innovative work-behavior.

Summarizing, our study strongly shows enhancing employees work-related flow through empowering leadership behavior to sustain innovative work-behavior during the COVID-pandemic. Directive leadership, in contrast, can reduce work-related flow and, therefore, hinder innovation. This outcome is an important contribution to the scholarly and societal debates on how to ensure innovative behavior, also during the COVID-19 pandemic (Montani and Staglianò, 2021).

### The Relation of Time to the Moderating Role of IT-Enabled Presence Awareness in the Relationship Between Leadership and Work-Related Flow

Regarding IT-enabled presence awareness, the results also showed no significant evidence of its moderating role in the relationship between leadership and work-related flow during the second measurement. This result is at odds with the extant literature that advocates the influence of IT-enabled presence awareness in a remote work context (Malhotra and Majchrzak’s, 2014; Lim, 2018). However, the companies in our study may have matured in their use of informational technology over the past years, and many employees, while being challenged with lower communication richness, were already familiar with the use of IT-facilitated communication (Garro-Abarca et al., 2021) before the COVID-19 pandemic. This may explain why IT-enabled presence awareness did not play a significant role in the relationship between leadership and work-related flow in both phases of the lockdown due to the COVID-19 pandemic.

Moreover, misalignment between employees that can be resolved by easier access to IT-solutions that enable clear communication often stems from different contexts in which individual employees operate and their knowledge thereof (Hinds and Weisband, 2003). Probably, respondents in our sample already had a shared context with their colleagues (Hinds and Mortensen, 2005), for which we did not control in our analysis.

### Limitations and Suggestions for Future Research

Despite its contributions, the present study was also subject to various limitations. First, while our cross-sectional research design sheds light on the relationships between our study’s core variables at two phases in the COVID-19 pandemic and changes herein, we did not investigate the causal relationships over time. While research on the long-term effects of leadership on innovative behavior during the pandemic is still ongoing (Bajaba et al., 2021), based on the results of our study, we encourage scholars to adopt similar or longitudinal research designs.

Second, our sample is heterogeneous and unbalanced in terms of occupational groups and industry. Therefore, the representability of our sample is limited. Future research could consider using a larger sample that is more balanced in respondents’ characteristics, which would increase their outcomes’ generalizability.

Third, regarding a possible moderating influence of IT-enabled presence awareness on the relationships between leadership, and work-related flow our study did not find significant relationships despite prior research ascribing an important role to the construct in home-based working (Majchrzak et al., 2000; Malhotra and Majchrzak’s, 2014; Lim, 2018). Decreasing communication richness during the COVID-19 pandemic, however, has been an important challenge within organizations (Garro-Abarca et al., 2021). Future studies could include and control more contextual factors that might explain employees’ perceptions regarding IT-enabled presence awareness, such as shared team contexts (Hinds and Mortensen, 2005), degree of familiarity between members prior to working from home (Gruenfeld et al., 1996), alternative IT channels through which leadership behavior can be conveyed (Lim, 2018), and differences in geographic contexts (Hinds and Weisband, 2003).

Fourth, while empowering and directive leadership are important leadership styles in both ‘normal’ contexts (Lorinkova et al., 2013) and during crises (Stoker et al., 2019), many more leadership styles could be studied. For example, shared leadership (Pearce and Sims, 2002) might be an interesting avenue for future research to discover how empowered teams develop leadership capabilities as they are provided with autonomy and support (Ahearne et al., 2005).

### Managerial Implications

Aside from previously discussed theoretical implications, our study also contributed knowledge that is relevant for practitioners. First, while it is understandable for leaders to tighten to leash and become more directive in their leadership behavior when employees are working remotely (Stoker et al., 2019), we encourage leaders to empower employees through support and autonomy instead. By focusing on empowering leadership in the beginning phases of working from home, leaders can bring employees to take up broader responsibilities by expressing confidence in their work, causing them to experience work-related flow more frequently and encourage innovative work-behavior.

Second, based on the increasingly imperative role of work-related flow in the relationship between leadership and innovative work-behavior as observed in this study, we recommend employees to focus on increasing work-related flow experience to fuel their long-term ability to generate, promote and implement novel ideas. According to Bakker and Van Woerkom (2017), employees can use four self-determination strategies to facilitate work-related flow experiences: self-leadership, job crafting, designing work to be playful, and focusing on using of their known strengths.

## Conclusion

This study unveiled the importance of leadership behavior to foster innovative behavior during the COVID-19 pandemic. By showing the positive direct relationship between empowering leadership and innovative work-behavior, and the negative direct relationship between directive leadership and innovative work-behavior, we shed more light on which (initial) leadership behaviors are most beneficial in a lockdown, such as the one caused by the coronavirus (Stoker et al., 2019; Bajaba et al., 2021). Moreover, our research underlined the importance of work-related flow in sustaining innovative work-behavior for employees who are working remotely. Initially, it showed that empowering leadership can foster work-related flow (Bakker, 2008) which can amplify innovative behaviors. Moreover, in the context of working from home during the COVID-19 pandemic, the importance of work-related flow in the relationship between leadership and innovative work-behavior only seemed to have increased after a prolonged time working from home. Empowered employees may have developed more confidence in their own abilities and autonomy and, therefore, work-related flow sustained their innovative behavior (Lorinkova et al., 2013). The mediating role of work-related flow also became stronger as it also mediated the negative effect of directive leadership on innovative behavior. As individual innovative work-behavior of employees, and their perceptions thereof (Janssen, 2000), are driving innovation on an organizational level (Liu et al., 2017), we enhanced our understanding of the influence of individual and organizational implications of the COVID-19 pandemic.

## Data Availability Statement

The raw data supporting the conclusions of this article will be made available by the authors, without undue reservation.

## Ethics Statement

Ethical review and approval was not required for the study on human participants in accordance with the local legislation and institutional requirements. The patients/participants provided their written informed consent to participate in this study.

## Author Contributions

All authors contributed to the conception and design of the study. MC and PP collected the data. PP conducted the data cleaning. MC, RE, and PP worked on the initial conceptualization of the research. RB did additional data-cleaning and analyzed the data in PLS-SEM. RE wrote the first draft of the manuscript. MC, PP, and RB supervised the study. All authors reviewed and edited the manuscript. All authors contributed to the manuscript revision and read and approved the submitted version.

## Conflict of Interest

The authors declare that the research was conducted in the absence of any commercial or financial relationships that could be construed as a potential conflict of interest.

## Publisher’s Note

All claims expressed in this article are solely those of the authors and do not necessarily represent those of their affiliated organizations, or those of the publisher, the editors and the reviewers. Any product that may be evaluated in this article, or claim that may be made by its manufacturer, is not guaranteed or endorsed by the publisher.
